# Quantification-based explainable artificial intelligence for deep learning decisions: clustering and visualization of quantitative morphometric features in hepatocellular carcinoma discrimination

**DOI:** 10.1117/1.JMI.12.6.061407

**Published:** 2025-10-11

**Authors:** Gen Takagi, Saori Takeyama, Tokiya Abe, Akinori Hashiguchi, Michiie Sakamoto, Kenji Suzuki, Masahiro Yamaguchi

**Affiliations:** a Institute of Science Tokyo, School of Engineering, Department of Information and Communications Engineering, Yokohama, Japan; b Keio University School of Medicine, Department of Pathology, Shinjuku, Japan; c International University of Health and Welfare, International School of Medicine, Narita, Japan

**Keywords:** artificial intelligence, image analysis, computer vision

## Abstract

**Purpose:**

Deep learning (DL) is rapidly advancing in computational pathology, offering high diagnostic accuracy but often functioning as a “black box” with limited interpretability. This lack of transparency hinders its clinical adoption, emphasizing the need for quantitative explainable artificial intelligence (QXAI) methods. We propose a QXAI approach to objectively and quantitatively elucidate the reasoning behind DL model decisions in hepatocellular carcinoma (HCC) pathological image analysis.

**Approach:**

The proposed method utilizes clustering in the latent space of embeddings generated by a DL model to identify regions that contribute to the model’s discrimination. Each cluster is then quantitatively characterized by morphometric features obtained through nuclear segmentation using HoverNet and key feature selection with LightGBM. Statistical analysis is performed to assess the importance of selected features, ensuring an interpretable relationship between morphological characteristics and classification outcomes. This approach enables the quantitative interpretation of which regions and features are critical for the model’s decision-making, without sacrificing accuracy.

**Results:**

Experiments on pathology images of hematoxylin-and-eosin-stained HCC tissue sections showed that the proposed method effectively identified key discriminatory regions and features, such as nuclear size, chromatin density, and shape irregularity. The clustering-based analysis provided structured insights into morphological patterns influencing classification, with explanations evaluated as clinically relevant and interpretable by a pathologist.

**Conclusions:**

Our QXAI framework enhances the interpretability of DL-based pathology analysis by linking morphological features to classification decisions. This fosters trust in DL models and facilitates their clinical integration.

## 1 Introduction

In recent years, the field of diagnostic pathology has seen a significant shift from conventional specimen analysis using light microscopes to digital pathology, where high-resolution digital images, such as whole slide imaging (WSI),^1^ are utilized for diagnostic purposes. Digital pathology is anticipated to play a significant role in remote diagnosis, the development of large-scale databases, and the establishment of more standardized diagnostic evaluations.^2^ In fact, it is already being utilized for applications such as immunohistochemistry scoring.^3^^,^^4^

Alongside this transformation in clinical practice, research has increasingly focused on leveraging deep learning (DL) technologies. Although the application of DL in clinical settings remains limited at present, a growing body of research has demonstrated its potential in carrying out various tasks relevant to digital pathology. For instance, self-supervised learning methods have been explored for feature extraction from unlabeled histopathological images, allowing the development of models that can generalize across different tissue types.^5^^–^^10^ Similarly, weakly supervised learning approaches, such as multi-instance learning (MIL), have shown promise in tasks such as tumor detection and classification using slide-level annotations.^11^^–^^14^ Advanced architectures, including graph neural networks (GNNs), have been utilized to model spatial relationships between cellular structures, providing insights into the tissue microenvironment.^15^^–^^18^ Domain adaptation techniques have further improved the robustness of DL models by addressing variations across staining protocols, scanners, and institutional data.^13^^,^^19^^,^^20^ In addition, the introduction of vision transformers has led to significant advancements in image-based feature extraction and classification tasks within pathology.^21^^–^^24^ Generative AI models have also been applied to data augmentation, stain normalization, and the simulation rare pathological images to enrich training datasets.^25^ Although these innovations have led to the development of highly accurate models, some of which have achieved diagnostic performance on par with—or even surpassing—experienced pathologists in specific research settings,^26^^,^^27^ their integration into routine clinical practice remains an ongoing challenge. Continued research and validation are essential to ensure their safe, effective, and ethically responsible deployment in real-world diagnostic workflows.^28^

DL is expected to enable high-accuracy inference based on an end-to-end approach, in which processing algorithm can be optimized by the learning of vast amounts of data. Thus, the DL–based process is essentially a black box. When the dataset size is limited, the algorithm can be partially hand-crafted, but the DL component remains nontransparent. If a pathologist receives results without any context or explanation, they may find it challenging to interpret or apply the information in their work. It is, therefore, imperative to elucidate the underlying basis for obtaining such inferential results.

Recently, extensive studies have been conducted on explainable AI (XAI) or interpretable DL.^29^^,^^30^ Medical image analysis is a field that particularly requires such techniques.^31^ Typical examples of XAI techniques are heatmaps,^8^ including GradCAM ^32^^,^^33^ and methods that highlight critical regions for discrimination, such as LIME.^34^ Therefore, most of the conventional XAI methods for DL achieve interpretation through visualization. This may be because conventional techniques were designed to provide interpretations for data analysts or system developers, whereas XAI specifically for pathologists is required in this field. XAI in pathology image analysis must provide pathologists with useful information assist in understanding the rationale behind the DL model’s inferences, and visualization alone is insufficient in achieving this.

Let us recall an important advantage of introducing digital image analysis in pathology: it allows for the quantitative description of morphological features providing measurement-based objectivity in diagnosis. Therefore, we focus on the issue that XAI for pathology should offer interpretations based on quantification: quantification-based XAI (QXAI).

In this study, we proposed QXAI to identify critical regions and their corresponding features quantitatively within the DL model’s discrimination process using hematoxylin and eosin (H&E)-stained pathology images of hepatocellular carcinoma (HCC). The aim is to clarify “which regions” and “what features” within the image contribute to the model’s discrimination, all while maintaining high inference accuracy. In the proposed method, clustering was conducted using DL embeddings, and clusters that significantly contributed to discrimination were selected. Furthermore, explainability was achieved by presenting the features of each cluster in a format that is easily interpretable by pathologists, utilizing handcrafted quantitative features. This approach allowed us to identify essential clusters for discrimination without sacrificing accuracy and to explain the model using objective and quantitative features. By bridging the gap between model explainability and accuracy, this method contributes to the advancement of AI-driven pathology research and lays the groundwork for developing transparent and clinically applicable diagnostic support systems.

## 2 Related Work

In pathology, explainability of DL discrimination is essential because diagnostic decisions directly influence treatment strategies and patient outcomes. Pathologists must be able to justify their conclusions to ensure that their recommendations to clinicians are both reliable and actionable. As prognostic evaluation plays a critical role in guiding treatment decisions, recent research has focused on extracting interpretable information that contributes to accurate outcome predictions.^35^

XAI has been advancing rapidly since originating from a DARPA project.^36^ Among various techniques, Grad-CAM^32^ has been extensively utilized in image processing. Grad-CAM visualizes important pixels for prediction outcomes based on gradient information in the convolutional layer of a convolutional neural network (CNN), and its application to pathological images has been documented.^33^ Similarly, in multiclass classification studies utilizing attention mechanisms, the heatmap technique has been employed to highlight regions critical to the prediction outcomes.^8^ Although these visualization techniques have proven valuable for highlighting important regions within input data, a more comprehensive understanding of XAI requires categorizing these methods based on their interpretability strategies and applicability across different model architectures.

XAI techniques can be categorized along several dimensions.^31^^,^^37^ One classification distinguishes between model-based and post hoc explanations. Model-based approaches enforce explainability during training, whereas post hoc methods seek to explain trained black-box models after their predictions are made.^31^ Another important distinction is between model-specific and model-agnostic explanations. Model-specific methods are designed for particular model architectures, whereas model-agnostic approaches work independently of the model structure, focusing only on input-output relationships. In addition, XAI frameworks differentiate between transparent models and post hoc explanations applied to black-box models.^37^ Transparent models are inherently interpretable but may compromise predictive performance, whereas post hoc techniques enhance the explainability of more accurate yet opaque models. This reflects a fundamental trade-off between accuracy and transparency, especially significant in medical applications where predictive performance often takes precedence.

XAI can also be categorized based on the intended audience. Visual explanation methods, such as Grad-CAM and heatmap, are primarily designed for developers and researchers, offering insights into the internal behavior of DL models by highlighting important regions associated with predictions. These methods are useful for model validation and debugging, but often fall short of providing clinically actionable insights. By contrast, explanations intended for end-users—specifically, pathologists—require greater clarity and clinical relevance. Techniques such as example-based explanations and text-based explanations offer more intuitive interpretations by presenting similar past cases^38^ or describing relevant features in natural language.^39^ These methods align more closely with clinical reasoning processes, facilitating trust and practical utility in diagnostic workflows.

MIL has emerged as a promising approach for analyzing WSIs. MIL enables the labeling of entire images as positive or negative while learning from segmented patches, allowing the identification of regions that significantly influence model predictions. This explainability makes MIL particularly suitable for applications where understanding the relationship between localized regions and overall diagnostic outcomes is essential.^12^^,^^13^^,^^22^ Reflecting this association in the image, methods that display important regions as a heatmap are being actively researched. Such heatmap-based methods^5^^,^^6^^,^^8^^,^^9^^,^^13^^,^^14^^,^^40^^,^^41^ are visually intuitive and enhance the explainability of DL models. However, understanding the specific characteristics of critical regions and the reason for their importance remains challenging through visualization alone.

There are also methods that utilize clustering as well as visualization. Several studies identify key features for each cluster while also generating heatmaps using unsupervised learning through clustering.^42^ Other studies involve pathologists labeling clusters obtained through clustering and subsequently relearning the data,^43^ or employing multivariate linear regression analysis to identify clusters significant for diagnosis, with the pathologists then describing the features of these clusters.^44^ These methodologies aim to enhance the explanability in diagnostic pathology by combining clustering and regression analysis to identify important features alongside visualization. However, these methods present several issues. They require pathologists to manually provide descriptions of the features and carry out cluster labeling, which is laborious and time-consuming. In addition, it is challenging to present accurate explanations in non-typical cases. Although it would be valuable to deliver explanations through texts or examples prepared by pathologists to adapt an XAI-based diagnosis support system into current practice, it is important to note that the primary advantage of computational pathology lies in its ability to facilitate the use of quantitative information on tissue morphological features, thereby supplementing traditional microscopic visual examination.

In this study, we propose a novel post hoc explainability framework tailored for black-box DL models applied to histopathological analysis. Our framework operates independently of the internal structure of a specific DL model, as it leverages latent feature embeddings and output predictions. Although our experiments employ only EfficientNetB3, the proposed method is applicable to other DL architectures as well. By integrating DL embeddings with quantitative, handcrafted features, the method offers objective explanations that are directly interpretable by pathologists. This approach uniquely identifies both the regions and features that contribute to model decisions without compromising accuracy.

## 3 Materials and Methods

### 3.1 Data

The image data utilized in this study were collected from the Department of Pathology at Keio University, Japan, and comprised H&E-stained histopathological specimens of HCC and non-neoplastic background (BG), digitized into WSIs at 20× magnification (μm/pixel) using a digital slide scanner. As the proposed method is designed for a general two-class discrimination problem, the labels positive (P) and negative (N) are used instead of HCC and BG to ensure generality in the following description. From the WSIs, regions of interest (ROIs) with a resolution of 2048×2048  pixels in an area of $∼1\;{\mathrm{mm}}^{2}$ were selected and annotated by pathologists at Keio University. This image size was selected to provide a field of view and resolution that were both necessary and sufficient for accurate P, N, and grade evaluations by the pathologists. Although fixed-size ROIs were used in this study due to dataset constraints, the proposed method is compatible with variable input sizes.

In addition, the images used in this study include both biopsy and surgically resected specimens, with the number of cases and images detailed in Table 1. Biopsy specimens, typically collected through needle biopsies prior to surgery to assess the lesions and the need for surgery, are relatively small and often contain a significant amount of background and fragmented tissues with artifact. Conversely, surgically resected specimens are larger in size and more morphologically preserved. The HCC images from the surgical specimens are annotated using Edmondson-Steiner classification,^45^ which histologically assesses the tumor grade and assigns a score ranging from 1 to 4. ROIs were selected so as not to mix different grades and cancerous/noncancerous areas as much as possible. Images classified within boundaries of each grade are assigned intermediate scores in increments of 0.5 (e.g., 1.5, 2.5, and 3.5), as shown in Fig. 1. The labeling of P and N, as well as the grade evaluations, were independently conducted by three pathologists. The distribution of images for each grade is presented in Table 2.

**Table 1:** Number of ROI images.

	HCC	BG	Number of total images (cases)
Surgical specimens	554	550	1104 (111)
Biopsy specimens	557	510	1067 (198)

BG, background; HCC, hepatocellular carcinoma.

**Fig. 1:**
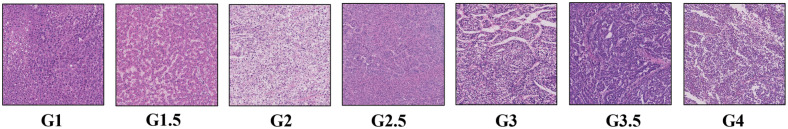
Examples of images from each grade.

**Table 2:** Number of images of surgical specimens in each Grade.

	Grade 1	Grade 1.5	Grade 2	Grade 2.5	Grade 3	Grade 3.5	Grade 4
Number of ROIs	82	33	289	49	62	9	23

ROI, region of interest.

All ROIs were divided into three subsets: training, validation, and test data, with a ratio of ∼3:1:1. To mitigate the risk of overfitting, images originating from the same case were consistently assigned to the same subset. The training subset was used for fine-tuning the CNN model, and the validation subset was utilized for hyperparameter optimization, as detailed in Secs. 3.3.2 and 3.3.3. In addition, the clustering process described in Sec. 3.4 employed the validation subset. The inference data (test subset in the experiment) was processed as detailed in Sec. 3.5 to produce the quantitative feature representation.

### 3.2 Overview of Proposed Method

The method proposed in this study aims to provide an objective basis for discrimination in DL using pathological images. It leverages clustering and handcrafted feature quantification while maintaining high accuracy in discrimination. Figure 2 presents an overview of our proposed method, which consists of three main sections: (a) training and clustering, (b) quantitative feature characterization, and (c) inference of new data. In panel (a), a DL model is first trained with patch images obtained by tiling labeled ROI images. After that, clustering is employed using the embeddings generated by this model. Then, clusters that contribute to discrimination are selected. In panel (b), the selected clusters are quantitatively characterized in terms of handcrafted features obtained by the feature selection using LightGBM. Section (c) involves the processing of new data, which allows for both high-accuracy discrimination and a presentation of the regions and features within the image that serve as the basis for discrimination, thereby enhancing the explainability of a DL-based diagnosis support system.

**Fig. 2:**
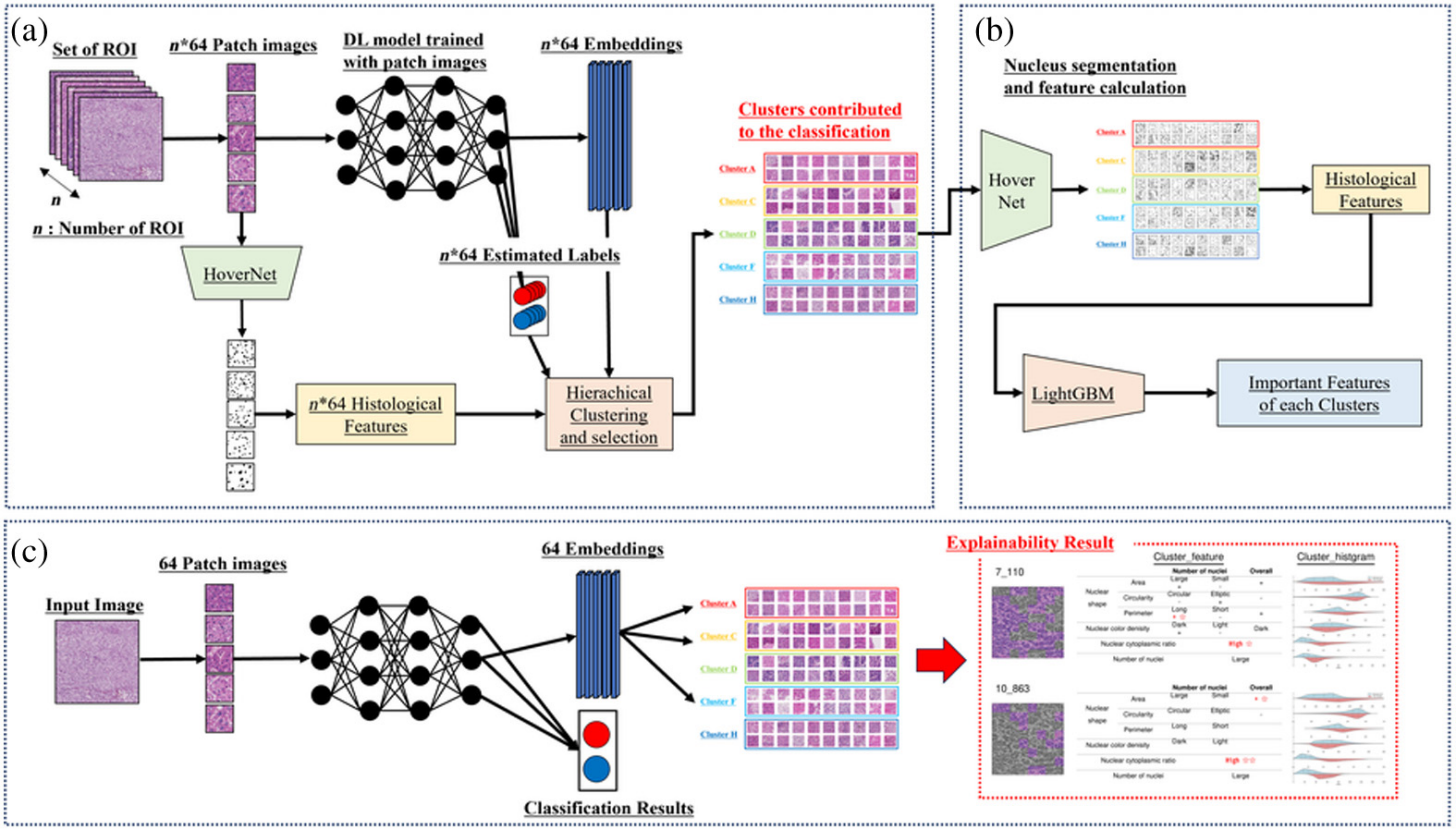
Overview of the proposed method: (a) Training and clustering: a set of labeled region of interest images of (H&E)-stained tissue is divided into small patches and used to train a deep learning (DL) model (EfficientNetB3). Hierarchical clustering is then performed using the embeddings obtained from the trained DL model and the inference results. The clustering process stops when almost all of the members in a cluster are correctly classified. The stopping criterion also considers the differentiability of the cluster using handcrafted morphological features obtained through nuclei segmentation with HoverNet. Finally, the clusters that consist almost exclusively of true positive or true negative samples are selectively retained as the clusters contributing to the discrimination. (b) Quantitative feature characterization: to elucidate the characteristics of these selected clusters, cell nuclei are segmented by utilizing HoverNet, and morphological features are calculated. Important features characterizing each cluster are selected using LightGBM, providing a quantitative explanation of the characteristics of each cluster. (c) Inference of new data with explained results. A new ROI image is input into the DL model, producing the discrimination results. Each patch is classified into one of the clusters generated in step (a). Based on the characteristics of the cluster corresponding to each patch, the explanation of the inference result is presented by colored regions that indicate whether the region contributes to the discrimination, along with tables and violin plots exhibiting quantitative representation of nuclei and tissue features.

### 3.3 Model Training

#### 3.3.1 Data pre-processing

The image data used in this study consists of a 2048-pixel square ROI. Due to the large size of the ROIs, direct input into a DL model is not feasible, and because fine details are critical in pathological images, resizing them to smaller dimensions is also inappropriate. Hence, each ROI is divided into 256-pixel square patch images. These patch images inherit the annotations associated with the original ROI. Some patch images contain mostly glass regions and lack any tissue content, and thus, they are excluded from the analysis. For this purpose, the patch images are first converted to gray scale and then binarized via Otsu’s method. Patches that indicate pixel intensity values above the preset threshold are classified as blank areas and are consequently excluded from both the training and inference phases of this study.

#### 3.3.2 Training using pathological images

In this study, binary discrimination between P and N is conducted using patch images. The model employed was EfficientNetB3,^46^ implemented via the timm^47^ library in PyTorch.^48^ Fine-tuning is carried out using initial weights derived from a model pre-trained on ImageNet. Only the training subset is used to update model weights, whereas the validation subset is used to compute the validation loss at the end of each epoch. The training process involves batches of 128, employing Adam as the optimizer (with a learning rate of $3.0\times 10^{-4}$ and a weight decay of $3.0\times 10^{-4}$), over a span of 100 epochs following PyTorch’s cosine annealing schedule. A termination strategy is in place during training, halting the process if validation loss fails to improve across successive epochs. This computation is executed on a TITAN RTX Graphics Card, equipped with 24 GB of VRAM.

#### 3.3.3 Discrimination of region of interest (ROI)

Although the DL model is trained using patch images, it is necessary to assess the entire ROI to verify its accuracy. In MIL, RNNs^12^ or transformers^12^ are typically employed to capture the correlations between individual tiles after feature extraction. However, due to the limited size of the dataset in this study, adequately training these correlations is complex. To address this, the following method is adopted.

For each ROI, 64 patch images are input into the learning model, and 64 outputs are global average-pooled to calculate a single value. For ROIs where patch images were removed during preprocessing, the score is adjusted by multiplying by a normalization factor (64/number of remaining patch images) based on the number of remaining images. The threshold used for classification is determined based on the validation subset to optimize discrimination performance. Finally, the scores are evaluated against a predetermined threshold. ROIs scoring above the threshold are classified as cancerous, whereas those scoring below are classified as non-cancerous. This method enables efficient discrimination of ROIs and WSIs, even when working with small datasets.

### 3.4 Clustering and Determination of Contributed Clusters

#### 3.4.1 Extracting histological features

Prior to the clustering phase, histological features were quantified to use in both clustering and explanations, with feature extraction occurring at the level of each patch image.

Initially, cell nuclei are extracted using HoverNet,^49^ followed by the computation of six nuclear features: nuclear area, nuclear perimeter, nuclear circularity, nuclear color density, number of nuclei, and nuclear cytoplasmic ratio. Measurements for area, perimeter, circularity, and color density were conducted for each nucleus, where color density was determined as the mean gray-scale value within the nuclear body, excluding the periphery.

The number of nuclei denotes the total number of nuclei within a patch image, although the nuclear cytoplasmic ratio, ideally representing the nucleus area relative to the surrounding cytoplasm, was approximately quantified as the proportion of the nucleus area to the total tissue-present area within the patch image, due to challenges in cytoplasm discrimination. These features were proposed based on the General Rules of Liver Cancer.^50^ However, cells exhibiting sub-threshold chromatin and area were classified as inflammatory and consequently excluded from the calculation of the feature values of the cell nuclei. Although nuclear count and cytoplasmic ratio are singular features per patch image, the remaining four features are contingent upon the number of nuclei, thereby adding complexity to data analysis. To address this, the four nucleus-level features (area, perimeter, circularity, and color density) were aggregated at the 25th, 50th, and 75th percentiles, resulting in 12 derived features. When combined with the two patch-level features—number of nuclei and nuclear cytoplasmic ratio—each patch image is represented by a total of 14 distinct features.

#### 3.4.2 Overview of clustering

The inference considered in this study is two-class discrimination; as an example, let us consider classes P and N. Our goal is to identify the features that are meaningful for discrimination. If the decision boundary in the latent space were simple, it would be possible to find specific features useful for the discrimination by analyzing the feature values that significantly separate the distributions of samples in classes P and N, for example, by representing the two classes through handcrafted features. However, it is difficult to find the specific features that contribute to discrimination using this method, as the DL generates a complicated decision boundary. If only a limited number of features are considered as a rationale for discrimination, the accuracy of the discrimination will be depleted (Fig. 3).

**Fig. 3:**
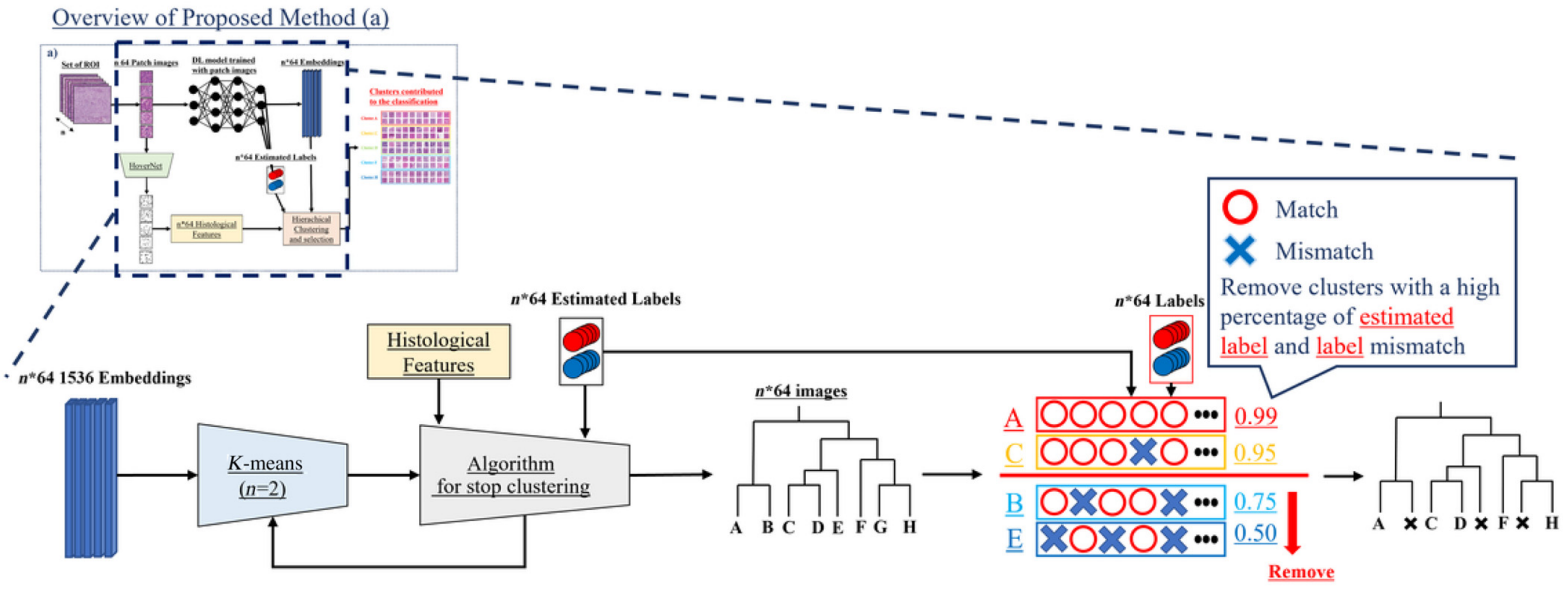
Overall clustering process: embeddings are obtained from the final layer of the deep learning model and employed for hierarchical clustering to generate a binary tree. Each node of the binary tree facilitates K-means clustering (K=2), with the stopping rule delineated by the algorithm presented in Fig. 4. From the resultant clusters, those comprising a high number of images with disparate inference and annotation labels are eliminated. This process leaves only the clusters that significantly contributed to the discriminant analysis.

Therefore, we apply a clustering technique to group similar samples in the latent space and analyze the features that differentiate each cluster. It will be possible to find the features that differentiate the cluster if it consists of similar samples in the latent space. By deriving useful features for each cluster, explanations can be provided without compromising accuracy, even in the case of complicated decision boundaries. The clustering is performed using only the validation subset, ensuring that the training data do not influence the feature interpretation process. To achieve efficient clustering without compromising accuracy, this method employs a top-down binary hierarchical clustering approach, as described in the next subsection:

#### 3.4.3 Binary hierarchical clustering

In the hierarchical clustering following a top-down binary tree approach, initially, all patch images are fed into the DL model trained as described in Sec. 3.3.2 to derive the output values of the fully connected layer, which are regarded as embeddings of patch images. The embeddings are subsequently utilized for k=2 K-means clustering. This process is iteratively repeated within each of the two clusters generated until the stopping criterion outlined in Fig. 4 is satisfied. The target for two-means is more than 50,000 images with 1536 embedding vectors, and the Rapids cudf^51^ library is utilized for fast processing in the experiment.

**Fig. 4:**
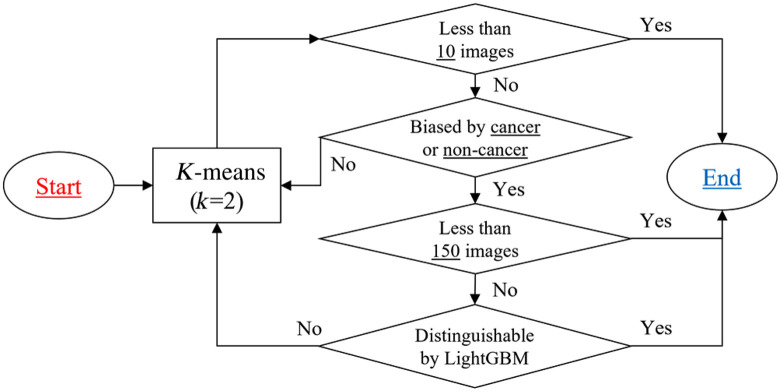
Criteria for stopping in the hierarchical clustering: the stopping criteria are as follows (images included in the clusters): less than 10 images, biased toward P or N, and less than 150 images, or more than 150 images and can be divided by LightGBM.

#### 3.4.4 Stopping criteria of clustering

The clustering process aims to identify groups of similar samples within the same class (P or N), where each cluster can be distinguished from others using quantitative, human-understandable features. Therefore, the stopping criterion for binary hierarchical clustering is based on whether a cluster consists almost exclusively of samples inferred as the same class. Because the feature space for clustering is derived from embeddings generated by the trained DL model, we utilize inferred labels rather than annotation labels to determine cluster purity. Annotation labels are independent of this feature space and do not directly reflect the structure imposed by the embeddings. To ensure that cluster division is halted when a sufficiently pure cluster is formed, we examine the inference output of the trained DL model and verify whether the predicted labels within a cluster exhibit a bias toward a single class that exceeds 95%. If this condition is met, further division of the cluster is stopped.

In addition, the cluster needs to be effectively distinguished using human-understandable features for the purposes of our QXAI. We employ LightGBM^52^ to assess whether the cluster can be classified using the handcrafted features, including the percentiles derived in Sec. 3.4.1, as explanatory variables. LightGBM is a gradient boosting-based decision tree algorithm renowned for its rapid training capabilities and high predictive accuracy. Moreover, it enables quantitative evaluation of each feature’s contribution to the loss function, thereby allowing explicit visualization of the features deemed important by the model during the inference process. LightGBM’s objective variable is set to 1 for patch images within the cluster and 0 for all others. The classification’s validity is determined by an F1 score of 0.9 or above. If this condition is satisfied, the clustering stops; otherwise, the cluster division is repeated, as shown in Fig. 4. This criterion is incorporated because when a biased and correctly classified cluster is large, samples with different features become mixed, making it difficult to explain the cluster’s features. Therefore, if the size of a cluster falls below a certain threshold, it is no longer divided and is determined as not contributing to discrimination.

#### 3.4.5 Cluster selection

The proposed method provides explanations using quantitative features for each cluster. However, not all clusters contribute equally to discrimination accuracy. Each sample processed for clustering corresponds to a patch image from an ROI, where the annotation label P or N is given to each ROI. Namely, the DL model is weakly supervised. Accordingly, some samples do not contribute to discrimination, such as patch images with tissue artifacts, image blurs, or no parenchymal cells included. These samples are classified randomly into classes P and N by the DL model, resulting in clusters that consist of non-contributing samples.

In ROI discrimination based on the DL model and clusters, all patch images obtained from the ROI are classified into clusters using a binary tree created through the hierarchical clustering step described above. Among these, only the DL model output from patch images classified into clusters that significantly contribute to discrimination is used to make a decision. To achieve this, we eliminate clusters with a high incidence of false inferences and select those with high discrimination accuracy.

To quantify cluster reliability, the error rate of each cluster is computed using validation samples by comparing annotation labels and model-inferred labels. Although the feature space for clustering is derived from DL embeddings and inference outputs, relying solely on inferred labels in this step would be inadequate, as it does not account for potential misclassifications made by the model. By incorporating annotation labels, we introduce a ground truth reference, allowing us to objectively evaluate whether a cluster predominantly contains correctly classified or misclassified samples. Clusters exhibiting elevated error rates are excluded through the iterative process described below.

The ROIs in the validation set are presented to the DL model, and the scores from the included patch images are aggregated to render a decision. However, in the proposed method, patch images from discarded clusters are omitted from this score compilation. In the first iteration, patch images from all clusters are used, except for images of blank areas and images belonging to small clusters (fewer than 10 samples). In the second iteration, patch images belonging to the cluster with the highest error rate are excluded. The accuracy for the entire ROIs is then systematically verified, and the cluster is deleted from the list of contributing clusters if the discrimination accuracy is almost equivalent to the case when all clusters are used. This exclusion continues until a noticeable decline in accuracy is observed. Ultimately, the retained clusters are those assessed as significantly contributing to discrimination accuracy. If the discrimination of ROI images using this clustering process achieves accuracy equivalent to the original DL model, it proves that the proposed XAI method provides an explanation while maintaining the accuracy of DL discrimination.

### 3.5 Quantitative Feature Representation

#### 3.5.1 Achieving explainability

After completing all training and clustering processes described above, the inference data are fed into the model, as illustrated in Fig. 2(c). The ROI is divided into 64 patch images, each of which is classified by the DL model. In addition to the classification results, the DL model outputs 64 embeddings for an ROI. Because a binary tree has already been constructed through hierarchical clustering, each embedding is assigned to a cluster by traversing the tree. Given that the binary tree is built using the k-means method with k=2, each embedding followed a path based on its proximity to the centroids at each split. By aggregating the clusters of all embeddings within the ROI, the “explainability result” shown in Figs. 2(c) and 5 can be generated.

**Fig. 5:**
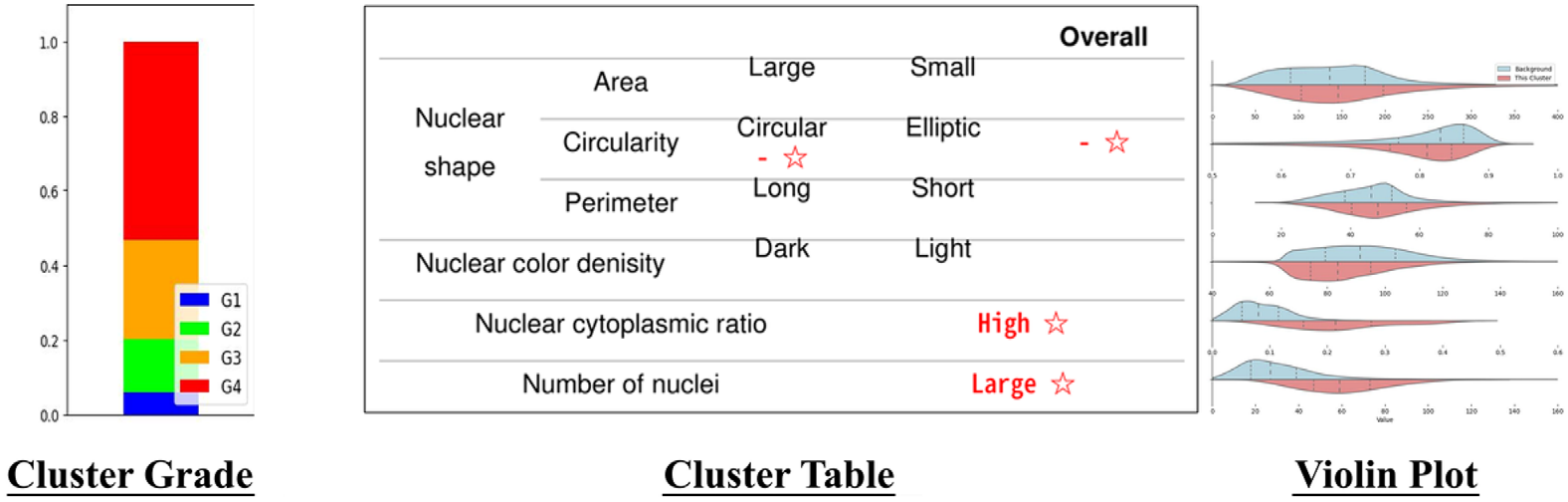
Explainability results based on reference grade labels and handcrafted features: this figure presents an example of the explanatory method detailed in Secs. 3.5.3 and 3.5.4, visualizing both the distribution of grades and the distribution of handcrafted features within a specific cluster. Left: the “cluster grade” represents the proportion of each malignancy grade present within the corresponding cluster. Center: the “cluster table” highlights the features identified by LightGBM as contributing to the classification, along with their respective importance scores. Right: the “violin plot” illustrates the distribution of each feature for the target cluster (red) in comparison to the BG (blue). Feature names on the vertical axis correspond to those in the cluster table, allowing for clear visual comparison of quantitative differences between features.

Even though discrimination accomplished by a DL model has a high level of complexity, the clusters obtained by the process depicted in the above sections are groups of similar samples, and the clusters can be characterized relatively easily using features understandable to humans. In the proposed method, the characterization of clusters is performed by handcrafted features, thereby elucidating the elements the DL model leverages for its classification decisions. Specifically, we incorporate four key elements: a cluster map, a cluster grade, a cluster table, and a cluster histogram. The following subsections provide a detailed explanation of each element.

#### 3.5.2 Cluster map

A cluster map shows areas that contribute to discrimination in an ROI, determined by clustering the patch images. The map has three categories: regions contributing to discrimination and inferred as class P, contributing regions and inferred as class N, and regions not contributing to discrimination. These categories are visualized using distinct colors: red and blue for regions inferred as P (HCC) and N (BG), respectively, and green for regions not contributing to discrimination. Color overlay is applied to enhance explainability; to preserve morphological detail, the original histological image is shown underneath the partially transparent color overlay. The category is determined by the type of cluster each patch image is classified into, with each category represented by a distinct color. This map visually represents which regions contribute to discrimination and whether they are classified as P or N. In addition, for clusters that contribute to discrimination, a map of the corresponding patch images is presented for each cluster, and the features within each cluster are depicted as described in the next subsections.

#### 3.5.3 Cluster grade

The task of this dataset is to classify P and N in the ROI. However, within P, each ROI is further annotated with a tissue classification grade: the Edmondson-Steiner grade, which reflects histopathological characteristics. To better characterize the clusters formed by our method, we introduce a cluster grade, which represents the percentage distribution of grades within a given cluster. This allows us to assess the predominant histological features associated with each cluster, providing additional interpretability into the model’s grouping of patches.

An alternative approach would be to develop a DL model specifically trained for direct grade classification. However, achieving high accuracy in such a model requires a significantly larger dataset with detailed, high-quality annotations. By contrast, our approach leverages existing grade annotations to offer an interpretable, data-efficient means of characterizing clusters without requiring additional labeled samples.

Cluster grade displays the percentage distribution of grades within the cluster. Figure 5 shows an example of a cluster grade, which shows a high-grade characteristic. As mentioned in Sec. 3.1, the annotations for grades include .5 values, leading to variation in the number of images for each grade. To address this issue, the .5 grades within a cluster are split equally, with half of the images being assigned to the adjacent integer grades, thereby normalizing the variation. (For instance, in a cluster with 30 total images, if there are 20 images of grade 2, 10 images of grade 2.5, and no images of grade 3, after normalization, there will be 25 images of grade 2 and 5 images of grade 3, with respective ratios of 25/30 and 5/30.)

Although cluster grade serves as a valuable reference for understanding the histological characteristics of clustered patches, it is important to note its inherent limitations. Because the grade labels are assigned at the ROI level, individual patch images within an ROI do not necessarily exhibit the same characteristics as the assigned grade. Thus, cluster grade should be interpreted as a supporting reference rather than an absolute determinant of patch-level histology. Nevertheless, this approach can be extended to other annotation types, particularly in cases where dataset size constraints prevent direct supervised classification using DL.

#### 3.5.4 Cluster table

The handcrafted features of the target cluster are presented in a tabular form, called a cluster table, as depicted in the center part of Fig. 5. The cluster table lists the 14 features in rows, where the descriptors “large,” “circular,” “long,” and “dark,” represent the 75th percentiles, and “small,” “elliptic,” “short,” and “light,” denote 25th percentiles for the area, circularity, perimeter, and nuclear color density, respectively. The trends of the 50th percentiles (median) are also presented under the label “Overall.”

The symbols (+, −, ±) indicate whether the number of nuclei in the key features of the target cluster is “increased,” “decreased,” or “unchanged” compared with those in all BG images. This assessment is conducted using the Wilcoxon rank sum test, which is a non-parametric statistical test (p value <0.05) that compares the distributions of two independent groups. For each feature, we compare its distribution in the target cluster against that of all BG images. For percentile-based features, the interpretation depends on whether the percentile reflects the upper or lower portion of the feature distribution:

• 75th percentile: A higher value means that larger feature values (e.g., nuclei with larger area) occur more frequently in the target cluster. In this case, “+” indicates an increase, and “−” a decrease relative to BG.
• 25th percentile: A higher value here means that the smaller feature values (e.g., nuclei with smaller area) are shifted upward and thus less frequent in the target cluster. Accordingly, such cases are marked as “−”. Conversely, when the 25th percentile is lower, it indicates that smaller feature values are more prevalent, which is marked as “+”. In other words, the symbols themselves retain their meaning (“+” = increased, “−” = decreased), but the intuitive interpretation flips: at the lower percentile, a higher value signals a reduction in small features, not an increase.

To illustrate, “elliptic” refers to the 25th percentile (first quartile) of the circularity distribution, whereas “circular” corresponds to the 75th percentile (third quartile). A small 25th percentile value implies many nuclei with small circularity (elliptic). Conversely, if the “+” or “−” symbol appeared under “circular,” it denotes a higher or lower proportion of circular nuclei contained in the patch image, respectively. Similarly, the symbols under “elliptic” indicate changes in the proportion of elliptic nuclei. Up to 14 features are thus expressed as “+”, “−”, or “±”, and features not deemed important are left blank.

Next, consider which features are important. Using the method implemented in Sec. 3.4.4, the target cluster was distinguished from other clusters using LightGBM with 14 features as input. LightGBM is particularly advantageous due to its ability to quantitatively assess the contribution of individual features to classification performance. In this section, we compute the feature importance for each cluster using this model and explicitly present the contribution of each feature. The importance values are normalized across the 14 features, ensuring that their cumulative sum equals 1. Furthermore, hierarchical annotations using “stars” are introduced to visually highlight highly contributory features, thereby facilitating intuitive identification of the most significant attributes. Specifically, features with an importance value exceeding 2/14, 4/14, and 6/14 are marked with one, two, and three stars, respectively. Because the average importance per feature is 1/14, these thresholds correspond to features with importance values that are at least 2, 4, and 6 times greater than the average, respectively. This tiered annotation provides a visually intuitive way of distinguishing features with significantly higher contributions. An example is shown in Fig. 5 for the cluster table.

#### 3.5.5 Cluster violinplot

Furthermore, Violin Plots enable a quantitative comparison of feature distributions both within and across clusters. The distribution of each feature in the target cluster and the corresponding distribution in all BG images are visualized using Violin Plots. In the figure, the target cluster distribution is depicted in red, whereas the BG image distribution is shown in blue. An example is provided in the right of Fig. 5. The vertical arrangement of the violin plots corresponds to the feature names listed in the cluster table, allowing for a visual and quantitative assessment of the degree to which each feature deviates from the BG distribution while simultaneously verifying the key features of the target cluster.

## 4 Experiments

### 4.1 Discrimination Accuracy

The proposed method divides data into clusters and uses the features of selected clusters to provide an explanation of discrimination. Before testing the explainability of the proposed method, we verified whether this cluster-based approach preserves the discrimination performance compared with the original DL model.

The training and clustering processes are conducted using five-fold cross-validation. In this scheme, each dataset is divided into five subsets: three folds are used for training, one fold serves as validation during training as well as for clustering, and the other fold is reserved for testing. This setup ensures that there is no overlap between the test data and the data used for clustering. During cross-validation, the assignment of training, validation, and test folds rotates across the five iterations, ensuring that each subset is used as a test set exactly once, whereas the others serve as training or validation folds. This procedure allows for a more robust evaluation by leveraging different portions of the dataset while maintaining strict data separation. To mitigate overfitting, samples from the same case are always assigned to the same fold, preventing data leakage across different partitions. In addition, each fold is balanced in terms of the ratio of P and N samples as well as the distribution of grades, ensuring that class imbalance does not introduce bias during training and evaluation.

To evaluate the effectiveness of the proposed method, we conducted four types of performance validation. First, we utilized an ImageNet-pretrained model, where only the fully connected layers were fine-tuned. Second, we trained a model exclusively on the dataset used in this study, without any pretraining. Third, we employed a transfer learning approach, initializing the model with ImageNet-pretrained weights and subsequently fine-tuning it on the training dataset. Fourth, we verified the accuracy of the proposed method using only the clusters that contribute to identification in the third method. The results are presented in Table 3. In addition, the proportion of clusters contributing to classification in the fourth method is presented in Table 4 for each fold. The number outside the brackets indicates the total number of clusters, and the number inside the brackets indicates the number of images included in all clusters.

**Table 3:** Performance comparisons of the CNN models (EfficientNetB3) trained in different scenarios in a five-fold cross-validation. Bold values indicate the best performance.

	Accuracy	AUC
All patches are used.		
Pretrained on ImageNet	0.926±0.007	0.927±0.007
Training from scratch	0.944±0.017	0.943±0.018
ImageNet transfer learning	0.978±0.006	0.979±0.006
Only patches of the contributed clusters are used.		
ImageNet transfer learning	0.975±0.005	0.973±0.008

**Table 4:** Number of clusters contributing to the classification in each fold in the five-fold cross-validation.

	All clusters	Contributed clusters	HCC	BG	Less than 10
One-fold	846 (23,414)	155 (19,338)	75 (9613)	80 (9725)	673 (3398)
Two-fold	838 (23,812)	130 (16,452)	76 (9532)	54 (6920)	669 (3377)
Three-fold	822 (23,624)	89 (11,444)	47 (6449)	42 (4995)	664 (3566)
Four-fold	897 (23,562)	127 (16,241)	67 (8631)	60 (7610)	719 (3640)
Five-fold	814 (23,327)	131 (17,078)	69 (9934)	62 (7144)	645 (3224)

Each value represents the number of clusters, whereas the numbers in parentheses indicate the total number of images contained within those clusters.

BG, background; HCC, hepatocellular carcinoma

From Table 3 comparing the discrimination accuracy of the ImageNet-pretrained model, the model trained from scratch, and the fine-tuned model, the transfer learning approach demonstrated the highest accuracy, underscoring the significance of both pre-training and dataset-specific fine-tuning. Furthermore, the model trained solely on the dataset outperformed the ImageNet-pretrained model, highlighting the effectiveness of pathology-specific learning. In addition, compared with previous studies using handcrafted features and support vector machine-based classification,^53^ our DL-based approach achieves significantly higher accuracy, further validating the advantages of feature extraction through deep neural networks.

Furthermore, in the scenario where only the contributed clusters were used, there was no significant decrease in accuracy compared with using all clusters. It confirms that selecting contributed clusters allows for efficient model building while maintaining accuracy. As shown in Table 4, the number of contributed clusters is significantly lower than the total number of clusters, and the number of images contained within these clusters is also reduced. Despite this reduction, Tables 3 and 4 demonstrate that the model maintains its classification performance. This demonstrates that contributed clusters can be effectively identified while maintaining classification performance. Table 4 also shows that the number of clusters varies significantly across different folds. Ideally, if the dataset were sufficiently large, clustering would yield a consistent set of clusters with similar counts across all folds. However, due to the limited dataset size in this study, variations in data distribution among folds likely led to fluctuations in the number of generated clusters. As the dataset size increases, this variability is expected to diminish, resulting in more stable cluster formation across folds.

### 4.2 Clustering Result

This section presents the characteristics identified through clustering. As shown in Table 4, the number of clusters varies across different cross-validation splits, making it impractical to report results for all folds. Therefore, for this analysis, we employ a separate data split that differs from the cross-validation scheme described in Sec. 4.1, utilizing a single-fold division instead. Specifically, the dataset is partitioned into training, validation (used for clustering), and test sets in a 3:1:1 ratio, ensuring that samples from the same case remain within the same subset to prevent data leakage. In addition, the distribution of P and N samples is balanced across the splits, and efforts are made to minimize Edmondson-Steiner grade^45^ imbalance. Unlike the five-fold cross-validation setup, where grade balance was not explicitly enforced, this single-fold approach allows for better control over grade distribution, which was previously infeasible due to data limitations preventing even distribution across all five folds.

As part of the clustering analysis, we employed LightGBM to evaluate whether individual clusters could be distinguished from the BG clusters based on handcrafted features. For clusters that could be successfully classified, we identified the most influential features using feature importance scores computed from the LightGBM model.

Tables 5 and 6 summarize the characteristic features of HCC-related and BG clusters, respectively, whereas Tables 7 and 8 present the six largest clusters in terms of the number of images contained. These tables provide an overview of how multiple morphological features are distributed within the largest clusters, illustrating their interrelationships rather than offering a fully quantitative analysis.

**Table 5:** Number of clusters per feature representation in HCC.

Feature	+	−	±	None stated
Area large	9	1	3	37
Area small	1	7	2	40
Area overall	14	2	2	32
Circularity circular	2	11	8	29
Circularity elliptic	13	0	5	32
Circularity overall	1	13	5	31
Perimeter long	12	1	1	36
Perimeter short	1	8	3	38
Perimeter overall	12	2	1	35
Nuclear color density dark	12	4	7	27
Nuclear color density light	2	7	7	34
Nuclear color density overall	2	7	7	32
	High/large	Low/small	Medium/normal	None stated
Nuclear cytoplasmic ratio	16	2	2	30
Number of nuclei	26	1	2	21

**Table 6:** Number of clusters per feature representation in BG.

Feature	+	−	±	None stated
Area large	6	5	5	37
Area small	6	7	3	37
Area overall	8	5	3	37
Circularity circular	8	4	8	33
Circularity elliptic	6	6	5	36
Circularity overall	8	7	4	34
Perimeter long	7	3	6	37
Perimeter short	3	7	6	34
Perimeter overall	8	6	3	36
Nuclear color density dark	5	7	7	34
Nuclear color density light	13	7	5	28
Nuclear color density overall	8	6	3	36
	High/large	Low/small	Medium/normal	None stated
Nuclear cytoplasmic ratio	9	10	3	31
Number of nuclei	12	13	6	22

**Table 7:** Number of clusters per feature representation in HCC.

Rank		1	2	3	4	5	6
Size		592	475	469	419	411	347
Cum. contributed		8.9%	16.0%	23.0%	29.3%	35.4%	40.6%
Area	Large	+	+	+		+	+
	Small	+	−	−			−
	Overall	−	+	+	+		
Circularity	Circular	−	−	−		±	
	Elliptic	+	+	+			−
	Overall	−	−	−	±	±	
Perimeter	Long	+	+	+			
	Short	±	−	−			
	Overall	±	+	+			
Nuclear Color density	Dark	+	+	+			
	Light	−	−	−		+	−
	Overall	−	−	−	−	+	
Nuclear cytoplasmic ratio		High	High	High	High	High	High
Number of nuclei		Large	Large	Large	Large	Large	Large

**Table 8:** Number of clusters per feature representation in BG.

Rank		1	2	3	4	5	6
Size		418	415	412	403	388	346
Cum. contributed		7.3%	14.6%	21.0%	28.9%	35.7%	41.7%
Area	Large	−	+	+	+	+	+
	Small	+	−	−	−		−
	Overall	−	+	+	+		
Circularity	Circular	−	+	±	+	−	−
	Elliptic	+	−	−	−	+	+
	Overall	−	+	+	±	±	
Perimeter	Long	−	+	+	±	+	−
	Short	+	−	−	−	−	+
	Overall	−	+	+	+	+	−
	Light	−	+	+	+	+	±
	Overall	±	+	+	+	+	+
Nuclear cytoplasmic ratio		Low	High	Low	High	Low	Low
Number of nuclei		Small	Large	Small	Large	±	Small

Tables 5 and 6 present the number of clusters classified under each feature category: “+”, “−”, “±” (plus-minus), and “None stated”. Here, “+” indicates that the feature is present in greater amounts, whereas “−” denotes a lower presence. The “±” category represents features that do not exhibit statistically significant differences, and “None stated” indicates that the feature was not identified as important by LightGBM. In addition, the term “Overall” in the feature description signifies the global assessment of the feature across the cluster—where “+” indicates a generally high presence and “−” indicates a generally low presence.

In Table 5, an increased nuclear count was identified as a key feature of HCC-related clusters. In addition, many clusters exhibited larger nuclear areas, irregular nuclear shapes, darker nuclear staining, and increased nuclear density. However, rather than being defined by individual features alone, these clusters are characterized by distinct combinations of morphological attributes, highlighting the ability of the proposed method to capture complex feature patterns rather than isolated descriptors. Table 7 further illustrates these relationships within the largest HCC clusters, providing insight into how multiple morphological features co-occur in high-frequency clusters.

Table 6 presents the characteristic features of BG clusters, which serve as the baseline for comparison. Although no highly distinctive features were observed, a subset of clusters exhibited lighter nuclear staining, suggesting minor deviations from typical BG characteristics. Table 8 provides additional insight into the largest BG clusters, demonstrating how various morphological features interact within these clusters.

In addition, Tables 7 and 8 contain blank fields, which indicate that certain features were not explicitly classified as important by LightGBM. This does not imply that these features are irrelevant, but rather that other features in the cluster were assigned significantly higher importance, making the blank features comparatively less influential in the classification model.

Furthermore, although Tables 5 and 6 present a simplified categorical representation using “+”, “−”, and “±” for ease of interpretation, the proposed explainability method enables a more detailed quantitative analysis through violin plots. These plots, described in Sec. 3.5.4, allow for a more nuanced evaluation of feature distributions across clusters, further enhancing the interpretability of the model’s decision-making process.

### 4.3 Analysis of Surgical and Biopsy Specimens

#### 4.3.1 Analysis of discrimination accuracy

The dataset analyzed in this study comprises both biopsy and surgical specimens.^53^ Biopsy specimens are typically small, whereas surgical specimens are much larger and encompass more information, making the latter more effective for preparing a rich dataset. In addition, these two types of specimens are collected through different methods and have distinct appearances. In this study, we investigated the effectiveness of integrating surgical specimens with biopsy data to enhance learning from both types of specimens, despite their considerable differences in terms of method of collection and appearance. Table 9 presents the accuracy validation results for models trained exclusively on surgical specimens, biopsy specimens, and a combined dataset including both types of specimens. The results indicate that when trained solely on biopsy specimens, the model exhibits lower accuracy in classifying surgical specimens. Conversely, when trained solely on surgical specimens, the accuracy for biopsy specimens decreases. However, when both specimen types are included in the training process, the model achieves improved performance across both categories, demonstrating enhanced generalizability.

**Table 9:** Comparisons of the performance of the proposed models with different training data.

Training data	Test data	Accuracy	Specificity	Sensitivity	AUC
Surgical	Surgical	0.941	0.898	0.982	0.940
Surgical	Biopsy	0.605	0.206	0.965	0.585
Biopsy	Surgical	0.554	0.083	1.000	0.542
Biopsy	Biopsy	0.967	0.990	0.947	0.969
Surgical and biopsy	Surgical	0.932	0.898	0.965	0.932
Surgical and biopsy	Biopsy	0.953	0.961	0.947	0.954

#### 4.3.2 Analysis of clustering

The observed improvement in discrimination performance when training on both surgical (A) and biopsy (B) specimens suggests two possible underlying mechanisms: (1) the model learns to distinguish A and B and adaptively captures their distinct features, or (2) the model identifies domain-invariant features that are shared across both specimen types. If the latter is not achieved, training on surgical specimens alone would not contribute to improving the classification performance of biopsy specimens.

To investigate whether the model learns shared discriminative features between A and B, we applied the proposed explainability method to analyze the learned feature space. The clusters included in this study are derived from the model trained using the proposed method and validated through five-fold cross-validation, as described in Sec. 4.1. Specifically, we examined the clusters that significantly contribute to classification and evaluated the extent to which they contained mixed samples from both A and B. Table 10 presents the number of contributed clusters, as well as the number of clusters containing both A and B samples, stratified by cancerous and non-cancerous categories. For each cluster, the total number of images is indicated in parentheses, maintaining consistency with previous tables. In addition, the table includes threshold values of 10%, 20%, and 30%, representing the proportion of A and B images within a cluster. These values indicate the minimum percentage of both sample types required for a cluster to be classified as mixed. Based on these findings, we conclude that the model effectively captures common discriminatory features across both specimen types, reinforcing the generalizability of the learned representations.

**Table 10:** Accuracy for the proposed model when changing the training images.

ALL	Contributed	Mixed (≥10%)	Mixed (≥20%)	Mixed (≥30%)
1173 (23,094)	103 (12,388)	22 (2133)	13 (812)	2 (33)

Each value represents the number of clusters, whereas the numbers in parentheses indicate the total number of images contained within those clusters. The “Mixed (≥10%)”, “Mixed (≥20%)”, and “Mixed (≥30%)” columns denote clusters in which both surgical and biopsy specimens constitute at least 10%, 20%, and 30% of the total images, respectively.

#### 4.3.3 Analysis of data size

Following on from our findings so far, we tested how much quantity of biopsy specimens needs to be added to the training set when there are already a sufficient number of surgical samples, to adapt to the biopsy domain, where only a small number of images are typically available. To address this issue, we fixed the volume of data for surgical specimens (with 42,000 patch images) and gradually increased the amount of training data for biopsy specimens. Then, we observed the minimum number of images required to accurately identify both types of specimens.

Figure 6 illustrates the change in accuracy as the number of biopsy specimens was increased. Even when no biopsy specimens were included, an accuracy of over 0.86 was achieved. When ∼8000 biopsy specimens were added to the 42,000 surgical specimens, the discrimination accuracy for both specimen types became equivalent. Furthermore, the discrimination accuracy for biopsy specimens reached saturation when their number increased to about 19,000, which corresponds to ∼45% of the surgical specimens.

**Fig. 6:**
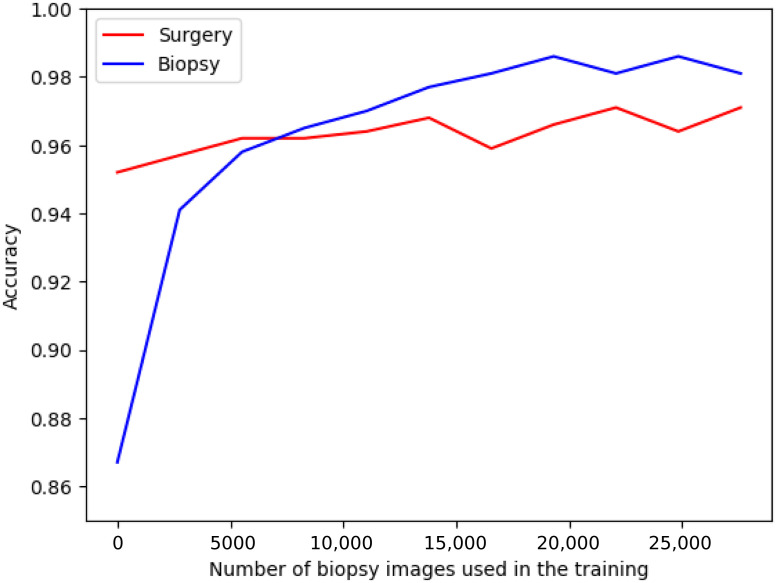
Change in accuracy over the number of training specimens (patches). The red line indicates the accuracy for the surgical specimens, and the blue line indicates the accuracy for the biopsy specimens. The vertical axis shows the accuracy.

These results suggest that a highly accurate model can be trained for both surgical and biopsy specimens even when the number of biopsy samples is limited, provided that a sufficiently large number of surgical specimens is available for training. Specifically, the accuracy for biopsy specimens becomes comparable to that of surgical specimens when they constitute ∼20% of the total dataset. This finding supports the feasibility of training an effective model for biopsy specimens using a relatively small sample size, which is particularly relevant given the challenges in collecting large biopsy datasets. However, it remains an open question how this trend generalizes to scenarios involving a significantly larger dataset with greater image diversity, including samples from multiple institutions, a broader range of histopathological conditions (e.g., benign lesions, precancerous changes), or pretraining on large-scale foundation models. Future studies should explore whether the observed saturation effect persists under such conditions and how the balance between surgical and biopsy specimens impacts model performance at an even larger scale.

### 4.4 Explainability of the Proposed Method

The proposed method was applied to 400 ROI images to discriminate between HCC and BG and to demonstrate how the model’s explainability works. As it is not feasible to present all images here, three representative examples are shown in Figs. 7–9. Figures 7 and 8 depict HCC images of surgical specimens, whereas Fig. 9 shows a BG image of a biopsy specimen.

**Fig. 7:**
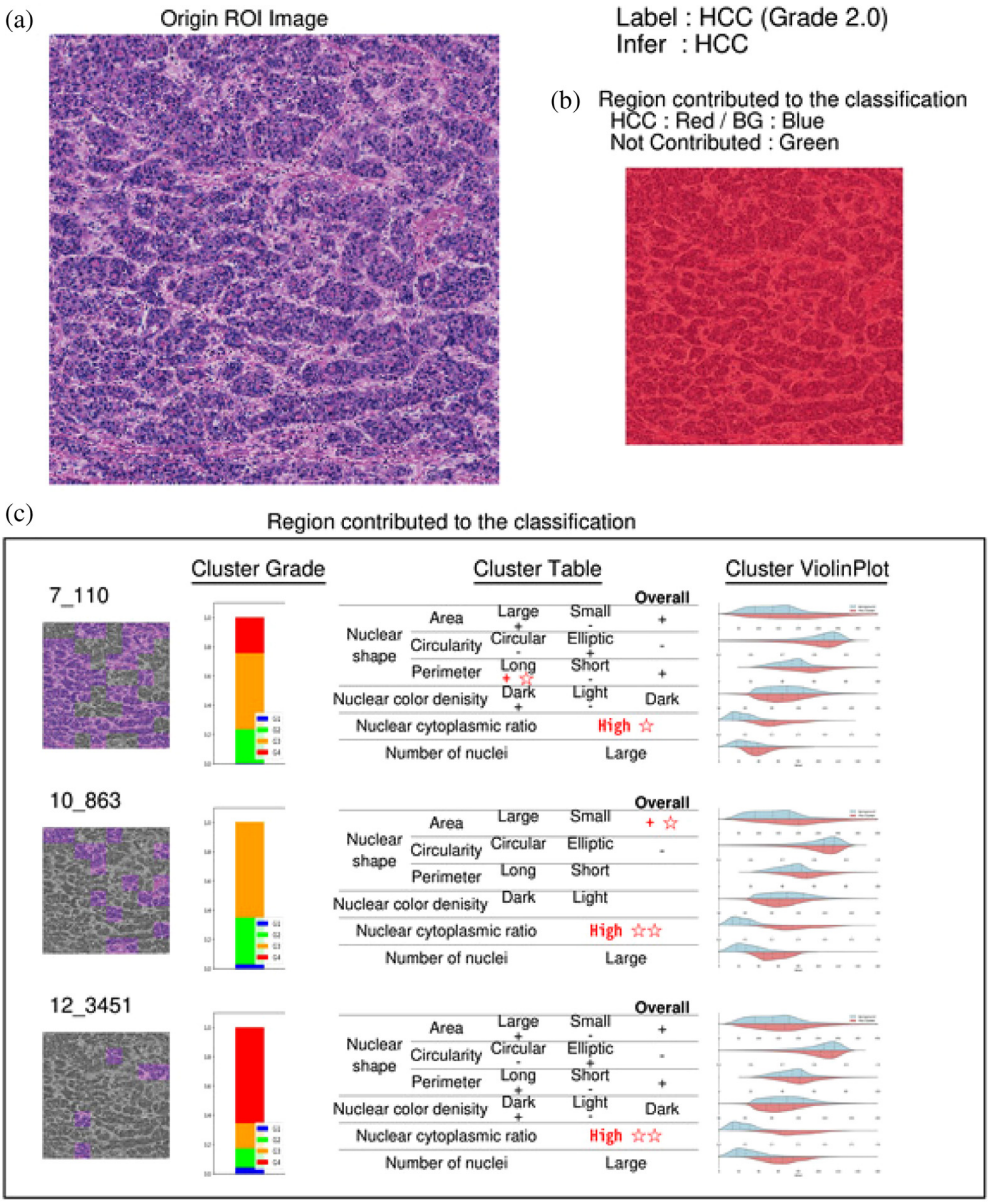
Results of the explainability experiment 1 (HCC [grade 2.0] images of surgical specimens) with our proposed method. This is a visualization of the application of the proposed method to images, along with the inference results and the insight into their explainability. (a) Input region of interest (ROI) image and (b) color-coded ROI image visualizing the regions that contributed to the discrimination of the classes: the contributed regions classified as hepatocellular carcinoma (HCC) are shown in red, the contributed background (BG) regions in blue, and regions that do not contribute to the discrimination in green. The details are described in Sec. 3.5.2. Because all regions are classified as HCC in this case, the entire image is overlaid in red. (c) The images highlight the regions that contributed to the discrimination by cluster and explain the characteristics of each cluster. The cluster grade indicates the percentage of malignancy in that cluster, and the cluster table and violin plot quantitatively illustrate the features of each cluster. The details are described in Secs. 3.5.3 and 3.5.4.

**Fig. 8:**
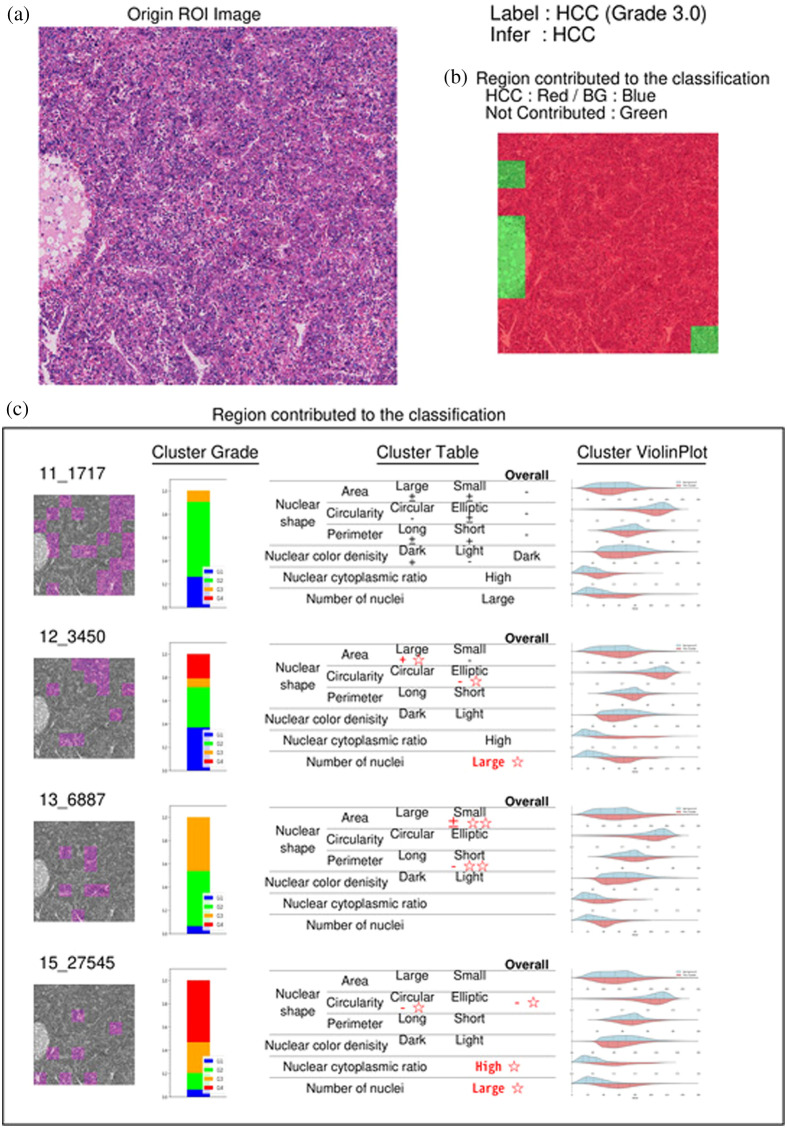
Result of the explainability experiment 2 (HCC [grade 3.0] images of surgical specimens) with method: (a) Input region of interest (ROI) image and (b) color-coded ROI image visualizing the regions that contributed to the discrimination of the classes. Because no region is classified as BG in this case, the entire image is overlaid only in red and green. (c) The images highlight the regions that contributed to the discrimination by cluster and explain the characteristics of each cluster.

**Fig. 9:**
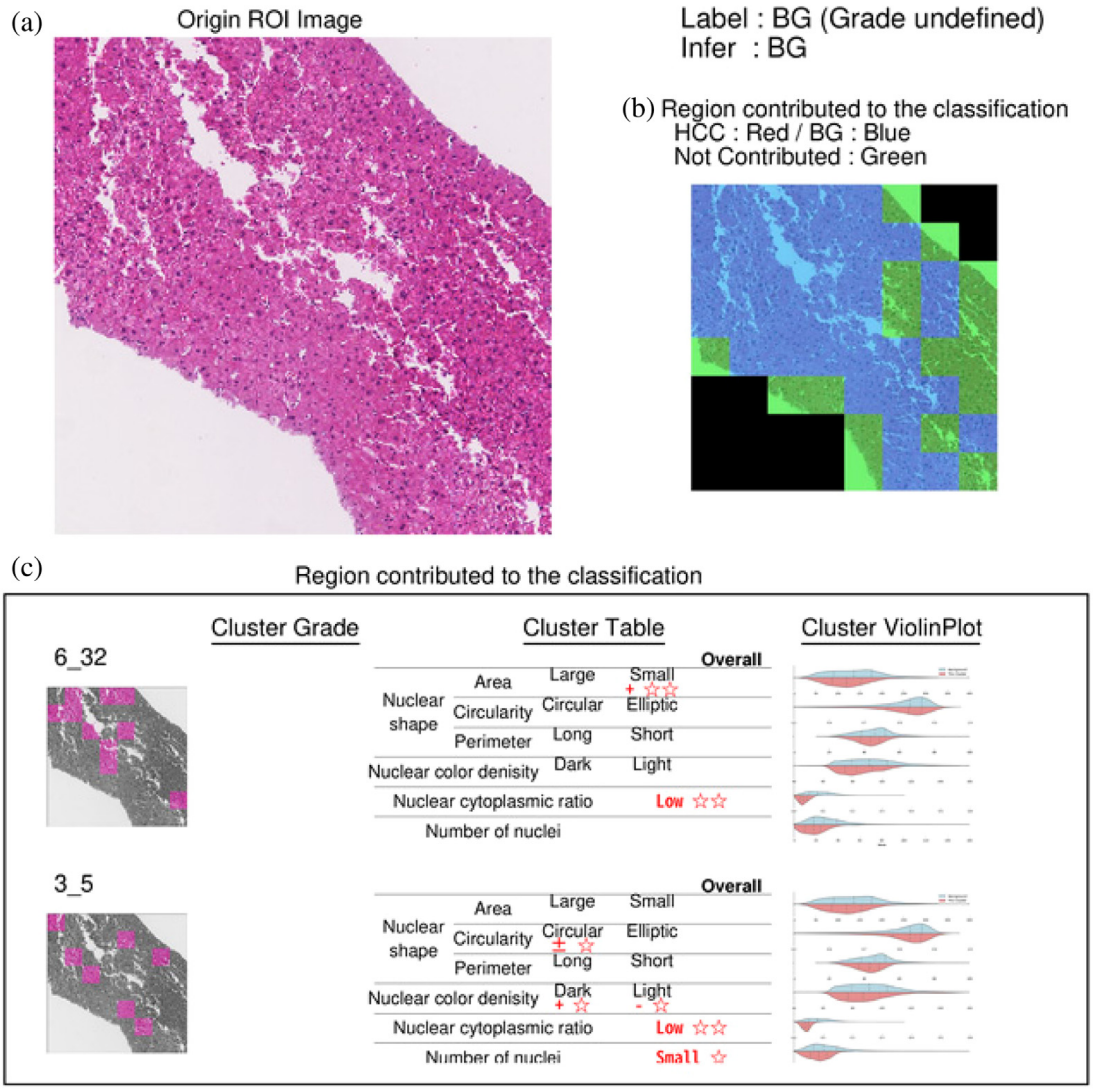
Result of the explainability experiment 3 (BG images of biopsy specimens) with method: (a) Input region of interest (ROI) image and (b) color-coded ROI image visualizing the regions that contributed to the discrimination of the classes. Because no region is classified as HCC in this case, the entire image is overlaid only in blue and green. (c) The images highlight the regions that contributed to the discrimination by cluster and explain the characteristics of each cluster.

In Fig. 7, all regions contribute to the discrimination and are further divided into three distinct areas. The cluster table and histogram results confirm that these three regions are markedly characterized by the nuclear cytoplasmic ratio. The largest region is also distinguished by a large perimeter, whereas the other two regions have even higher nuclear cytoplasmic ratios.

In Fig. 8, the left and lower right regions are identified as areas that do not contribute to discrimination, appearing to be interstitial. These regions are not typically considered by pathologists during diagnosis, indicating that they are correctly determined as non-contributory. Characteristically, compared with Fig. 7, the nuclear cytoplasmic ratio is not particularly large, there are fewer oval-shaped nuclei, and there are more areas of high nuclear density.

Figure 9 shows a BG image of the biopsy specimen, unlike Figs. 7 and 8. As is common in biopsy images, regions not used for discrimination are shown in black. Compared with the overall features of the background tissue, these regions exhibit a much smaller nuclear cytoplasmic ratio.

As described above, applying this method allowed us not only to show the discrimination results but also to clearly indicate the features of the regions contributing to discrimination using handcrafted features.

## 5 Conclusion

This paper proposes a novel explainability framework for DL-based pathological image analysis. The proposed method involves hierarchical clustering of feature embeddings, followed by the selection of discriminative clusters based on their contribution to classification. Handcrafted quantitative features are then extracted from these clusters, enabling interpretability by highlighting “which regions” and “what features” drive the model’s decision-making. By leveraging both model-derived representations and human-interpretable features, this approach enhances the explainability of DL models in pathology.

To evaluate the effectiveness of this method, we conducted experiments using H&E-stained histopathological images of HCC, consisting of both biopsy and surgical specimens. Model performance was assessed across different training configurations, and the results demonstrated that integrating both specimen types improved overall classification accuracy while revealing common discriminative features across domains. In addition, our approach provided a structured and quantitative way to interpret the model’s decision process, making it more suitable for clinical applications.

By extracting clusters that contribute to discrimination and expressing the handcrafted features of these clusters, this study was able to clarify the specific regions and features in the image that contribute to the model’s discrimination. This method enhanced the explanatory power of DL for pathological image diagnosis using objective and quantitative features.

However, several challenges remain with this study. One key issue is the limited number of human-interpretable features used in this study. There are many additional morphological features that could be considered, such as nuclear arrangement, cytoplasmic size, and the presence or absence of fatty degeneration. Another limitation is the expressiveness of the cluster features. A more effective representation method is needed to facilitate easier comparison between clusters.

## Data Availability

We shared the code of the proposed method on https://github.com/gen1998/qxai.The data are not publicly available.
